# Analysis of the impacts of climate change, physiographic factors and land use/cover on the spatiotemporal variability of seasonal daily mean flows in southern Quebec (Canada)

**DOI:** 10.1007/s13201-024-02180-9

**Published:** 2024-04-25

**Authors:** Ali A. Assani

**Affiliations:** Department of Environmental Sciences and the Research Centre for Watershed-Aquatic Ecosystem Interactions (RIVE, UQTR), University of Quebec at Trois-Rivières, 3351 Boulevard des Forges, Trois-Rivières, QC G9A 5H7 Canada

**Keywords:** Seasons, Seasonal daily mean flows, Rainfall, Temperature, Snowfall, Southern Quebec

## Abstract

The objective of this study is to compare the spatiotemporal variability of seasonal daily mean flows measured in 17 watersheds, grouped into three homogeneous hydroclimatic regions, during the period 1930–2023 in southern Quebec. With regard to spatial variability, unlike extreme daily flows, seasonal daily mean flows are very poorly correlated with physiographic factors and land use and land cover. In fall, they are not correlated with any physiographic or climatic factor. In winter, they are positively correlated with the rainfall and winter daily mean maximum temperatures. In spring, they are strongly correlated positively with the snowfall but negatively with the spring daily mean maximum temperatures. However, in summer, they are better correlated with forest area and, to a lesser extent, with the rainfall. As for their temporal variability, the application of six different statistical tests revealed a general increase in daily mean flows in winter due to early snowmelt and increased rainfall in fall. In summer, flows decreased significantly in the snowiest hydroclimatic region on the south shore due to the decrease in the snowfall. In spring, no significant change in flows was globally observed in the three hydroclimatic regions despite the decrease in the snowfall due to the increase in the rainfall. In fall, flows increased significantly south of 47°N on both shores due to the increase in the rainfall. This study demonstrates that, unlike extreme flows, the temporal variability of seasonal daily average flows is exclusively influenced by climatic variables in southern Quebec. Due to this influence, seasonal daily mean flows thus appear to be the best indicator for monitoring the impacts of changes in precipitation regimes and seasonal temperatures on river flows in southern Quebec.

## Introduction

Global warming and the resulting changes in temperature and precipitation regimes have a significant impact on the water cycle in general and river flows in particular. With regard to river flows, attention has been mainly focused on changes in extreme flows considered to be the most sensitive to global warming (e.g., Andreas et al. 2017; Berghuijs et al. 2014; Satoh et al. 2022). Nevertheless, some studies have also focused on detecting the signal of this warming by analyzing monthly and/or seasonal daily mean flows in several regions of the world (e.g., Arnell et al. 2015; Arrieta-Castro et al. 2020; Berghuijs et al. 2014; Langat et al. 2017; Lauro et al. 2019; Lobanova et al. 2018; Yao et al. 2020). Most of these works have clearly demonstrated that these flows can be just as sensitive to global warming as are extreme flows.

In Canada, there are still very few studies on the temporal variability of monthly and seasonal flows across the country (Coulibaly and Burn 2005; Nalley et al. 2012; Zhang et al. 2001) compared to those already devoted to extreme flows. A few rivers in the province of Quebec were analyzed as part of these studies. However, these rivers do not represent the obvious regional hydrological differences that exist in this province. These regional hydrological differences were taken into account in the only study devoted to the analysis of the variability of seasonal spring–summer and summer–autumn flows during the period 1950–2000 in relation to that of precipitation and climatic indices (Assani et al. al. 2012). This analysis of the temporal variability of flows was based on the linear regression. However, this method does not take into account the effects of short (STP) and long (LTP)-term persistence on the stationarity of the hydrological series. In addition, this study did not analyze the physiographic and climatic factors that influence the spatial variability of seasonal flows in Quebec.

Moreover, studies on the climate impacts caused by global warming have revealed that in Quebec, these impacts result in a significant drop in the amount of snow (Brown 2010; Guerfi et al. 2015; Yagouti et al. 2008) but by an increase in temperature and rainfall (Assani et al. 2019; Yagouti et al. 2008) since the middle of the last century. But the impacts of these changes in precipitation and temperature regimes on the seasonal daily mean flows of four seasons have never been studied.

To fill these gaps in knowledge of the spatiotemporal variability of seasonal flows in Quebec over a relatively long period, this study proposes to analyze the following aspects:

1. Compare the physiographic and climatic factors that influence the spatial variability of seasonal daily mean flows in Quebec. This objective is based on the following hypothesis: the spatial variability factors of seasonal flows differ according to the cold (winter and spring) and warm (summer and autumn) seasons due to the decrease in the amount of snow in the cold season on the one hand, and the increase in the amount of rain in the warm season on the other hand.
2. Compare the temporal variability of seasonal daily mean flows during the four seasons. The hypothesis underlying this objective is as follows: the impacts of changes in precipitation and temperature regimes on seasonal flows differ in cold seasons and in warm seasons. Due to the preponderant influence of the snowfall on seasonal flows (Assani and Tardif 2005), the reduction in this amount of snow will have a much greater impact on flows in cold seasons (winter and spring), generated mainly by snowmelt, than in warm seasons (summer and fall).
3. Determine which season is most impacted by changes in precipitation and temperature patterns.
4. Determine if the seasonal daily mean flows are as sensitive to changes in precipitation and temperature regimes as the extreme maximum and minimum daily flows in southern Quebec.

It is important to remember that this study is part of a study program which aims to determine the factors of spatiotemporal variability of flows on daily (maximum and minimum), seasonal and annual scales in the context of current global warming. The province of Quebec is one of the provinces in Canada which is bearing the brunt of the impacts of global warming which very significantly affect river flows. The monetary costs caused in particular by floods and hydrological droughts since 2008 have far exceeded billions of US dollars (e.g., Assani 2024). These changes in extreme flow regimes can affect seasonal daily average flows to varying degrees. These flows also play a crucial role for the ecological functioning of river ecosystems and their managements. Likewise, they can be just as sensitive to climate change as extreme flows. Hence, the need to analyze them.

After the introduction, the article presents the description of the study region, the variables analyzed and the statistical methods applied. This methodological part is followed by the parts devoted to the results and the discussion. It ends with a general conclusion.

## Methods

### Description of rivers and data source

**Fig. 1:**
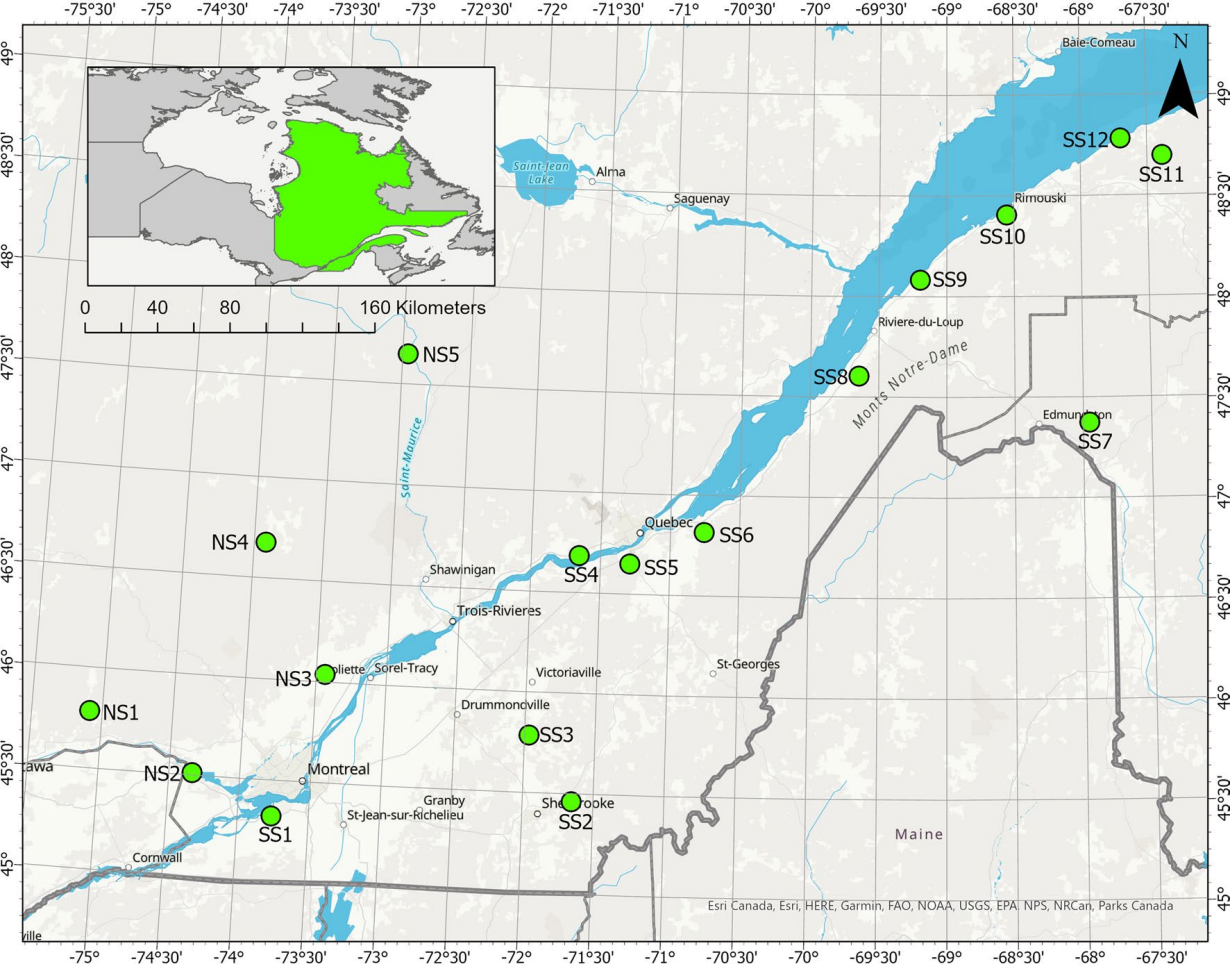
Location of stations. *SS* South Shore, *NS* North Shore

Seventeen rivers were selected for this study. They have been grouped into three homogeneous hydroclimatic regions already defined by Assani et al. (2012) using principal component analysis of seasonal flows and precipitation (Fig. 1 and Table 1). The choice of these rivers is justified by the existence of almost continuous measurements of daily flows (less than five years of missing data for 5 rivers) during the period 1930–2023. These are the longest flow measurements for a greater number of rivers in Quebec. In addition, they make it possible to analyze the impact of long-term persistence on the stationarity of hydrological series. This aspect is important for assessing the impacts of climate change on flows. In addition, these daily flow measurements are not affected by dams or reservoirs.

**Table 1:** Some characteristics of watershed analyzed

Rivers	Code	ID	Drainage area (Km^2^)	Latitude (°N)	Longitude (°W)
*Southeastern hydroclimatic region*					
Châteaugay	SS1	30905	2492	45°19′′49′′	73°45′44′′
Eaton	SS2	30234	646	45°28′05′′	71°39′18′′
Nicolet SW	SS3	30101	562	45°47′30′′	71°58′05′′
Beaurivage	SS4	23303	1152	46°39′25′′	71°39′18′′
Etchemin	SS5	23401	708	46°39′25′′	71°17′20′′
Du Sud	SS6	23106	821	46°49′22′′	70°45′22′′
*Eastern hydroclimatic region*					
Ouelle	SS7	22704	796	47°22′52′′	67°57′14′′
Du Loup	SS8	22513	1042	47°36′43′′	69°38′41′′
Trois-Pistoles	SS9	22301	930	48°05′21′′	69°11′43′′
Rimouski	SS10	22003	1615	48°24′46′′	68°33′18′′
Matane	SS11	21601	1655	48°46′25′′	67°32′25′′
Blanche	SS12	21702	223	48°47′20′′	67°41′51′′
*Southwestern hydroclimatic region*					
Petite Nation	NS1	40406	1331	45°47′27′′	75°05′22′′
Du Nord	NS2	40110	1163	45°31′08′′	74°20′11′′
L’Assomption	NS3	52219	1286	46°02′45′′	73°26′19′′
Matawin	NS4	50119	1387	46°40′50′′	73°55′00′
Vermillon	NS5	50142	2662	47°39′20′′	72°57′44′′

Two hydroclimatic regions are located on the south shore of the St. Lawrence River: the eastern hydroclimatic region, north of 47°N, and the southeastern hydroclimatic region, south of this parallel. The rivers of the first region flow over two geological formations: the Appalachians and the Lowlands of Saint-Laurent. These two geological formations are made up of sedimentary rocks covered with sedimentary deposits of different origins (marine, fluvial, glacial, etc.). Topographically, the Appalachians are an old, folded and eroded mountain range, while the Lowlands are almost zero-slope sedimentary deposits. The watersheds of the rivers of the Southeastern Hydroclimatic Region are almost entirely confined to the Lowlands, although the rivers originate in Appalachia. The climate is temperate oceanic north of 47°N but mixed south of this parallel. The third hydroclimatic region of the southwestern is located on the north shore. The rivers there mainly drain the Canadian Shield, a geological formation made up of metamorphic and magmatic rocks also covered with sedimentary deposits of various origins. The climate is cold temperate continental.

Data on physiographic characteristics and type of land use and land cover were measured by the Glaciolab laboratory of the University of Quebec at Trois-Rivières according to the methodology already described in detail by Kinnard et al. (2022) in particular. With regard to the wetland data, they were supplemented by those extracted from the database of Belzile et al. (1997) whose methodology has been described in detail by Assani (2022a) in particular. It is important to specify that the surface areas of wetlands include those of other types of water bodies and small lakes. Daily flow data were taken from the Center d’Expertise Hydrique du Ministère (CIEQ) website of the Ministry of the Environment and the Fight against Climate Change of the Province of Quebec (https://www.cehq.gouv.qc.ca/index_en.asp, accessed on 2024–01–28). As for the climatic data, they were extracted from the website of the Canadian Federal Ministry of Environment and Climate Change. These climatic data are averages of the climatic normals of monthly temperatures and precipitation calculated over the periods 1941–1970, 1971–2000 and 1981–2010 (https://climat.meteo.gc.ca/climate_normals/index_f.html, accessed on 2022–02–28). The physiographic, land cover and land use, and climatic variables are presented in Table 2.

**Table 2:** Correlation coefficients calculated between physiographic variables and magnitude (L/s/km^2^) of winter and spring daily mean specific flows from 1930 to 2023

Variables	Winter	Spring
*Physiographic variables*		
Drainage density (km/km^2^)	0.0658	0.4567*
Mean slope (m/km)	0.2567	0.1251
Forest surface area (%)	− 0.2322	0.1853
Agricultural surface area (%)	0.3197	0.0884
Wetlands surface area (%)	− 0.2187	− 0.3891
*Climatic variables*		
Winter/spring total rainfall (mm)	0.5650**	− 0.2973
Winter/spring total snowfall (cm)	− 0.0211	0.6412**
Winter/spring total precipitations (mm)	0.2351	− 0.0708
Winter and spring total rainfall (mm)		− 0.4380*
Winter and spring total snowfall (cm)		0.7406**
Winter and spring total precipitations (mm)		0.3320
Winter daily maximum temperature (°C)	0.5297**	
Spring daily maximum temperature (°C)		− 0.7884**
April daily maximum temperature (°C)		− 0.8191**
May daily maximum temperature (°C)		− 0.7737**
Winter-Spring daily maximum temperature (°C)		− 0.8084**

** = Statistically significant values at the 5% level; * = statistically significant values at the 10%; Bold = positive correlation; Italic = negative correlation

### Constitution of hydrological series and statistical analysis of data

#### Constitution of the hydrological series of seasonal daily mean flows

To constitute the series of seasonal average daily flows, the 12 months of the year have been grouped into the following four seasons: winter (January to March), spring (April to June), summer (July to September) and autumn (from October to December). The first two seasons (winter and spring) constitute the cold period because the flows of these two seasons come mainly from snowmelt and the temperatures are generally low. The last two seasons (summer and autumn) constitute the warm period because their flows come mainly from rain when temperatures are relatively high. However, in autumn, we can record the snowfall in November and December following the drastic drop in temperature. Nevertheless, the melting of this snow can occur in autumn but its contribution to fall flows is less important than that of rain. For each of four seasons and for each river, three-month daily flow averages were calculated for each year during the period 1930–2023. However, in autumn, the data analyzed stops at the year 2022. The data for 2023 were not yet available for all the rivers during data processing.

#### Statistical analysis of the spatial and temporal variability of flows

The spatial analysis of the flows consisted of comparing the average daily seasonal flows calculated over the entire study period (1930–2019) using parametric (ANOVA) and nonparametric (Kruskal–Wallis) tests. This comparison made it possible to determine regional differences in flows. Then, these averages were correlated with land cover, land use, physiographic and climate variables using linear correlation (parametric test) and Spearman rank (nonparametric test) methods.

With regard to temporal variability, the objective of this analysis is to verify the stationary or non-stationary character of the hydrological series in order to be able to detect the signal of the changes that have affected the temperature and precipitation regimes in the context of global warming in southern Quebec. This analysis was therefore carried out in the following three steps:

- In the first step, the classical Mann–Kendall (MK) test was applied to test the stationarity of the hydrological series. It was described by Sneyers (1990) in particular. But this test does not eliminate the effects of persistence, neither short-term (STP) nor long-term (LTP), which can affect the hydrological series and thus lead to erroneous conclusions on their stationarity.
- In the second step, we therefore applied four different tests that eliminate the effects of short-term persistence. These are the following tests: the Prewhitenning method (MMK-PW, Von Storch 1995), the Trend Free Prewhitening Method (TFPWMK, Yue et al. 2002), the Modified Mann–Kendall Test1 (MMKY, Yue and Wang 2004) and Modified Mann–Kendall Test2 (MMKH, Hamed and Rao 1998). The first two tests eliminate the effects of autocorrelation by the process of pre-filtering (prewhitening) of data and the last two by the correction of variance of the series.
- In the last step, to eliminate the effects of long-term persistence, the test proposed by Hamed (2009) (MK-LTP) was applied. This leads to the same results as that based on the Bayesian method. It is important to note that no spatial autocorrelation was observed between stations (Douglas et al. 2000). To avoid making the text unnecessarily heavy, the mathematical equations of these different tests will not be presented here because they are developed in the cited references.

## Results

### Comparison of the spatial variability of seasonal average daily flows

**Fig. 2:**
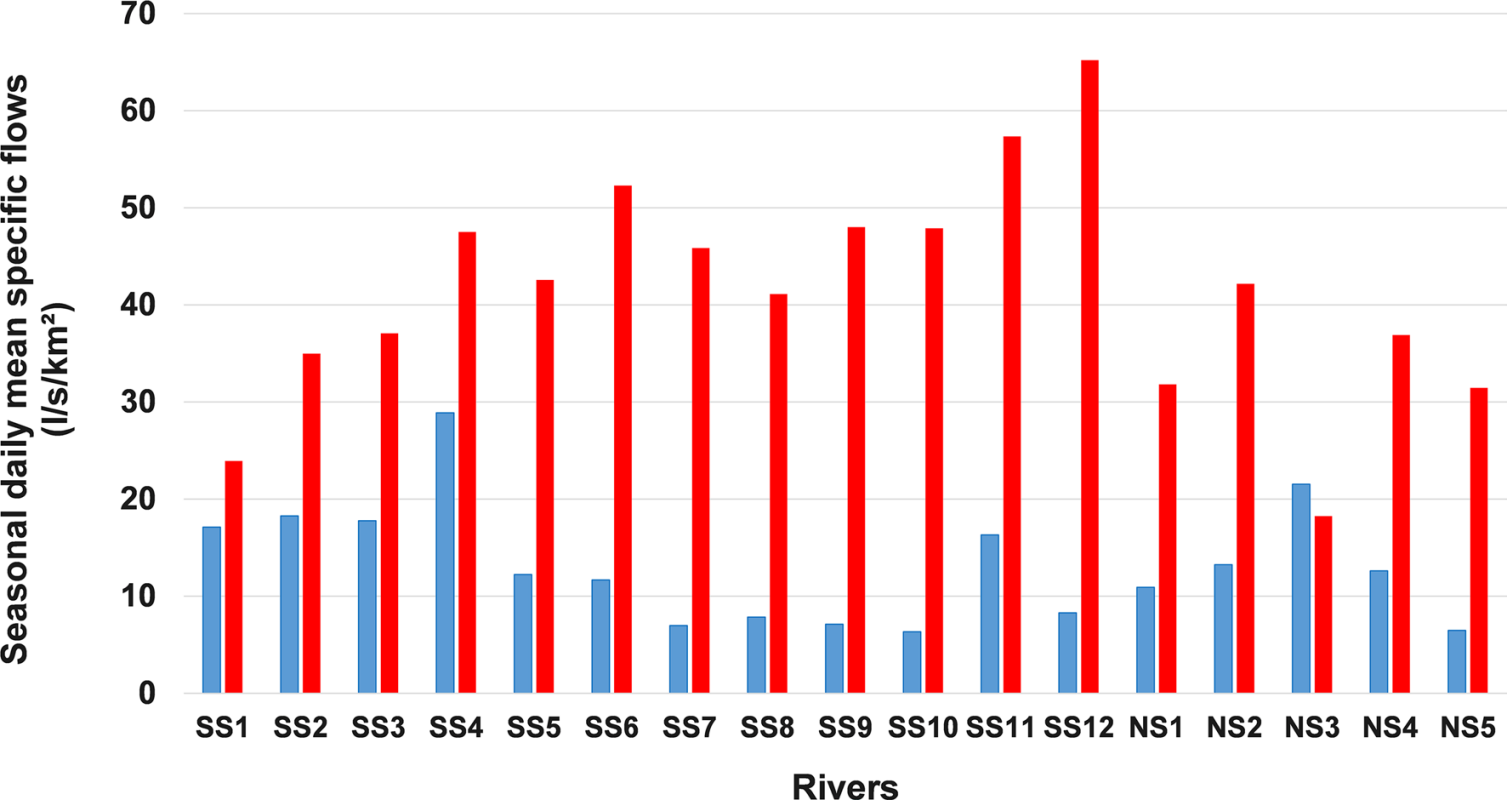
Comparison of averages of seasonal daily mean specific flows (l/s/km^2^) in the three hydroclimatic regions (1930–2023) in winter (blue bars) and spring (red bars)

The values of seasonal daily mean flows, expressed in specific flows, are presented in Figs. 2 (winter and spring seasons) and  3 (summer and autumn seasons). In winter, the application of ANOVA and Kruskal–Wallis tests revealed a significant difference in the means between the three hydroclimatic regions across southern Quebec. In fact, in the hydroclimatic region of the southeastern, the average flow values generally exceed 15 l/s/km^2^. This value is rarely exceeded in the other two hydroclimatic regions. The Eastern hydroclimatic region, located north of 47°N on the south shore, is generally characterized by the lowest flow values (< 10 l/s/km^2^), with the exception of the Matane River (SS11). As for the hydroclimatic region of the southwestern, located on the north shore, the value of 15 l/s/km^2^ was exceeded in the watershed of the L'Assomption River, which is the most agricultural and the most urbanized on this shore. It follows that the highest values of winter daily mean flows are observed in the most agricultural watersheds. This difference in average flows was also observed in the spring. However, unlike the winter season, there are no very clear regional differences (Fig. 2). Therefore, this statistically significant difference in the means detected is simply the result of the differences between certain rivers. For example, the average spring flows of the Matane River (SS11) are almost twice as high as those of the Châteaugay River (SS1), two rivers located on the south shore on either side of 47°N.

**Fig. 3:**
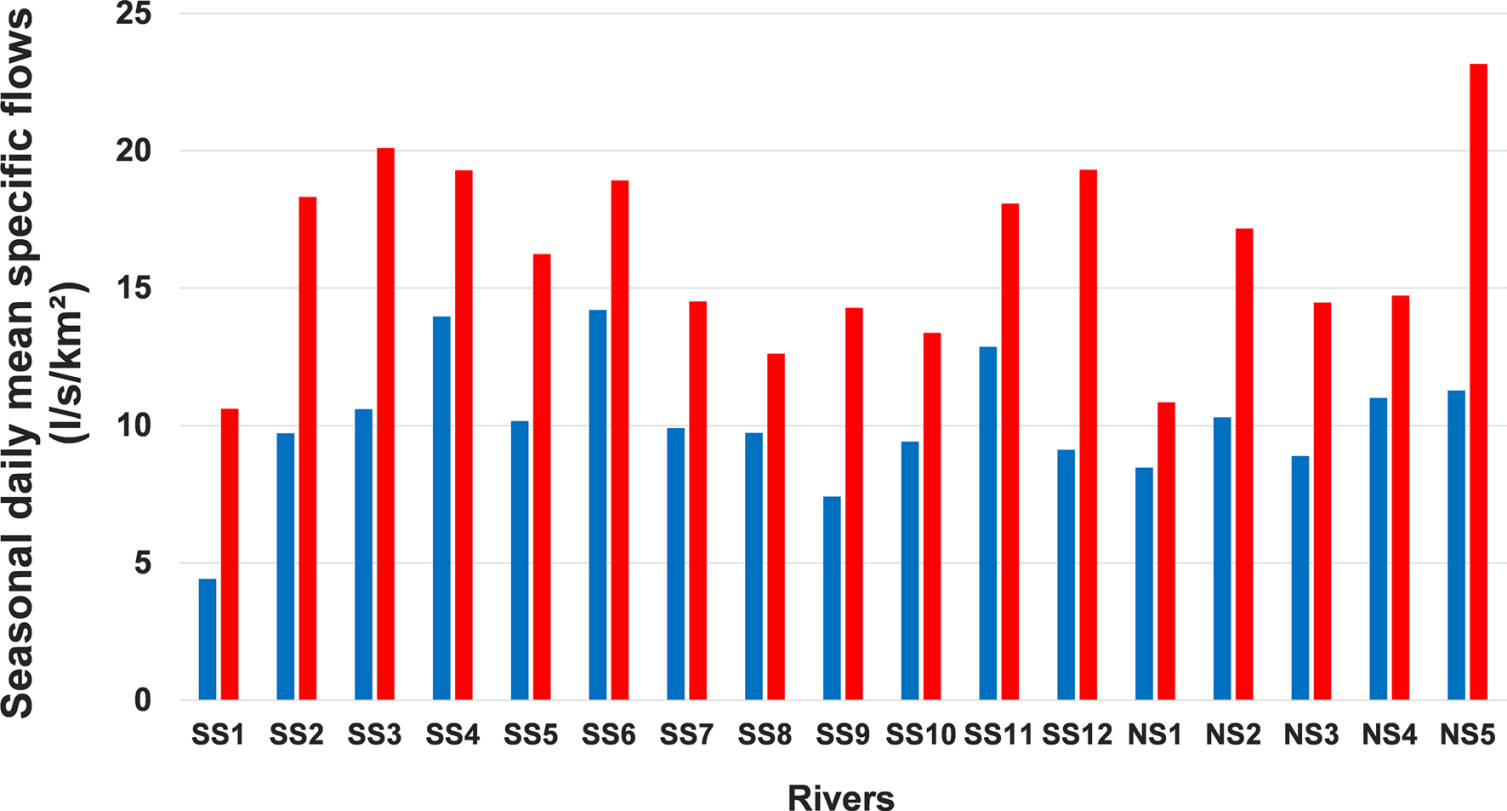
Comparison of averages of seasonal daily mean specific flows (l/s/km^2^) in the three hydroclimatic regions (1930–2023) in summer (blue bars) and fall (red bars)

Regarding the warm period, in summer, the application of these two above-mentioned tests also showed an overall difference in the averages of 17 rivers analyzed. However, there are no clear regional differences between the three hydroclimatic regions. Nevertheless, we can observe that in the Eastern hydroclimatic region, the value of 10 l/s/km^2^ was exceeded only in a single watershed, that of the Matane River. For all the other rivers in this hydroclimatic region, this value is below this threshold (Fig. 3). The same behavior is also observed in autumn.

### Comparison of factors of the spatial variability of flows during the four seasons

The values of the correlation coefficients are presented in Tables 2 (winter and spring) and  3 (summer and autumn). In winter, seasonal flows are positively correlated with the rainfall and winter daily mean maximum temperatures. They are not correlated to any physiographic factor. On the other hand, in spring, the flows are positively correlated to the drainage density. But this correlation is significant only at the 10% level. In addition to this factor, these flows are relatively strongly positively correlated with the snowfall in winter and spring on the one hand, but negatively correlated with the winter–spring daily mean maximum temperatures, in particular those measured in April, the first months of the spring season, on the other hand. Finally, spring flows are negatively correlated to the rainfall. But this correlation is significant only at the 10% level.

**Table 3:** Correlation coefficients calculated between physiographic variables and daily mean specific flows (l/s/km^2^) in summer and fall from 1930–2023

Variables	Summer	Fall
*Physiographic variables*		
Drainage density (km/km^2^)	0.3533	0.1232
Mean slope (m/km)	0.5226**	0.2685
Forest surface area (%)	0.5656**	0.2658
Agricultural surface area (%)	− 0.3122	− 0.0204
Wetlands surface area (%)	− 0.1989	− 0.1776
*Climatic variables*		
Summer total rainfall (mm)	0.4759*	
Fall total rainfall (mm)		− 0.2343
Fall total snowfall (cm)		− 0.0251
Fall total precipitations (mm)		− 0.1361
Summer-fall total rainfall (mm)		0.1008
Summer-fall total precipitations (mm)		0.0526
Summer daily maximum temperature (°C)	− 0.1037	
Fall daily maximum temperature (°C)		− 0.0837

** = significant correlation coefficient at the 5% threshold. Bold = positive correlation; * = significant correlation coefficient at the 10% threshold

During the warm period, in summer, summer daily mean flows are positively correlated with the average slopes of the watersheds and the area covered by forests. They are also positively correlated with the total summer rainfall. In autumn, the flows are not significantly correlated with any physiographic or climatic factor (Table 3).

### Comparison of the temporal variability of flows during the four seasons

**Fig. 4:**
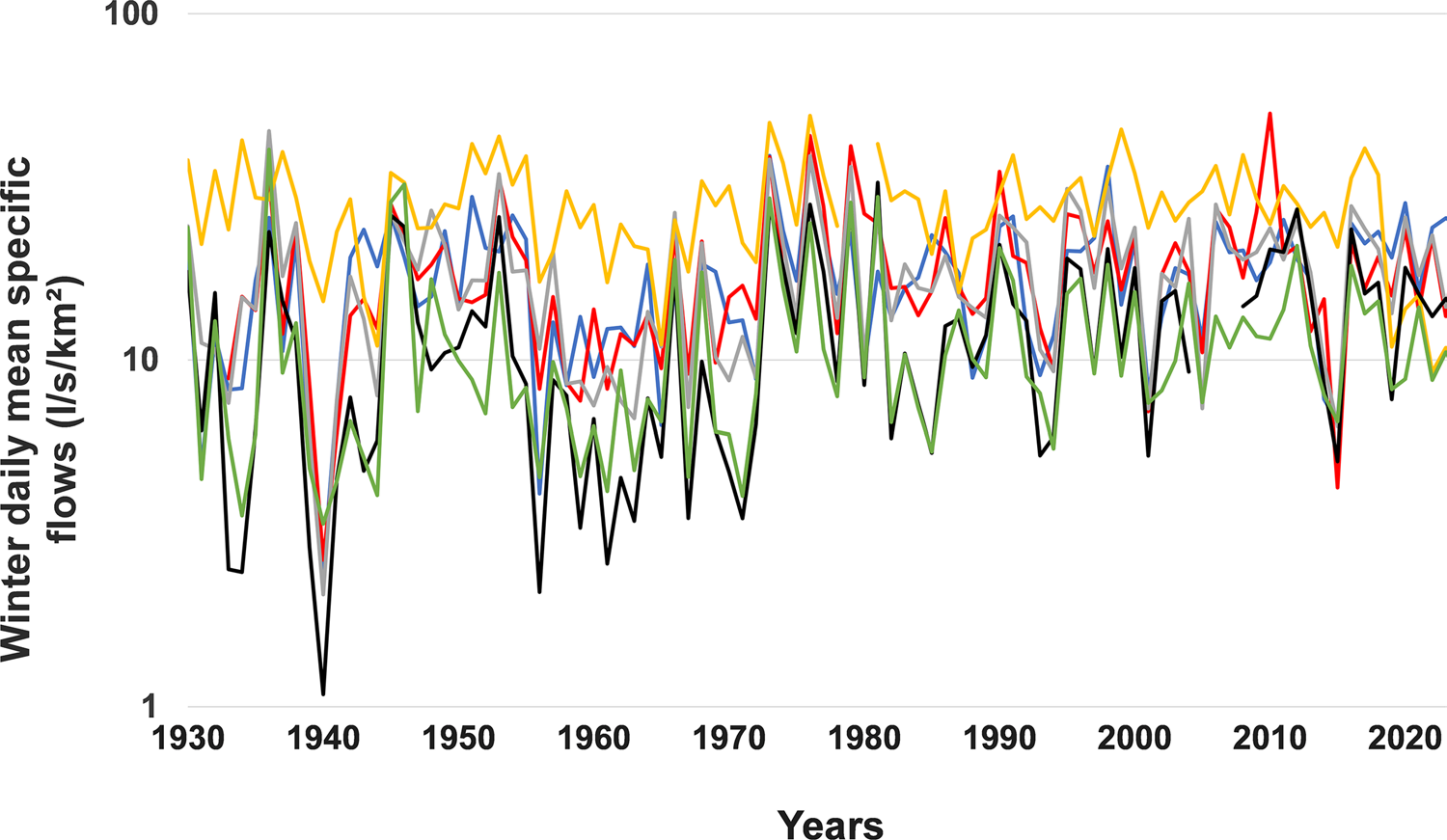
Interannual variability of winter daily minimum specific flows (l/s/km^2^) in the southeastern hydroclimatic region in south shore from 1930–2023. Châteaugay River (SS1, Blue curve), Eaton River (SS2, red curve), Nicolet SW River (SS3, gray curve), Etchemin River (SS4, yellow curve), Beaurivage River (SS5, black curve), Du Sud River (SS6, green curve)

The results obtained by applying six different Mann–Kendall statistical tests are presented in Tables 4, 5, 6, 7. In winter, the results of these six tests are almost consistent in the Eastern hydroclimatic region on the south shore to the north of the 47°N and in the southwestern hydroclimatic region on the north shore. In fact, these tests reveal a general increase in flows over time in these two hydroclimatic regions (Fig. 4). In the southeastern hydroclimatic region located on the south shore south of 47°N, the results of all the tests that eliminate short-term persistence (STP) effects are also consistent and also reveal a general increase in flows in this hydroclimatic region. On the other hand, this increase was detected only for two rivers by the test which eliminates the effects of long-term persistence (LTP).

**Table 4:** Results of the application of the various Mann–Kendall tests to the series of winter daily mean flows during the period 1930–2023

Rivers	MK		MMK-PW		TFPW		MMKY		MMKH		LTP	
	Z	*p* value	Z	*p* value	Z	*p* value	Z	*p* value	Z	*p* value	Z	*p* value
*Southeastern hydroclimatic region*												
Chateaugay	1.736*	0.090	1.534	0.125	1.853*	0.064	5.154**	0.000	2.014**	0.044	1.060	0.289
Eaton	2.090**	0.020	1.746*	0.081	1.992**	0.046	5.329**	0.000	1.962**	0.049	1.326	0.185
Nicolet SW	1.896*	0.077	2.052**	0.040	2.116**	0.034	5.297**	0.000	1.896*	0.058	1.456	0.145
Etchemin	1.336	0.182	1.496	0.135	1.719**	0.086	3.644**	0.000	2.007**	0.045	0.765	0.444
Beaurivage	3.016**	0.003	2.721**	0.007	3.283**	0.001	7.822**	0.000	3.603**	0.000	1.915*	0.056
Du Sud	2.858**	0.007	3.165**	0.002	3.105**	0.002	5.320**	0.000	2.681**	0.007	2.373**	0.018
*Eastern hydroclimatic region*												
Ouelle	1.768*	0.091	1.950*	0.051	2.029**	0.042	5.092**	0.000	1.768*	0.077	1.272	0.203
Du Loup	2.900**	0.002	2.846**	0.004	3.172**	0.002	8.036**	0.000	2.900**	0.004	1.924*	0.054
Trois-Pistoles	2.969**	0.005	3.264**	0.001	3.094**	0.002	9.160**	0.000	3.278**	0.001	2.523**	0.012
Rimouski	3.325**	0.003	3.441**	0.001	3.264**	0.001	12.79**	0.000	3.333**	0.001	3.370**	0.001
Matane	3.356**	0.000	3.182**	0.001	3.211**	0.001	10.61**	0.000	3.289**	0.001	9.494**	0.000
Blanche	3.827**	0.000	2.311**	0.021	3.594**	0.000	4.674**	0.000	2.443**	0.000	1.901*	0.057
*Southwestern hydroclimatic region*												
Petite Nation	4.872**	0.000	3.611**	0.000	4.965**	0.000	12.95**	0.000	5.582**	0.000	3.685**	0.000
Du Nord	3.115**	0.004	2.779**	0.005	2.851**	0.004	9.014**	0.000	3.500**	0.000	2.466**	0.000
L’Assomption	2.321**	0.010	2.087**	0.037	2.442**	0.015	5.800**	0.000	2.321**	0.000	1.554	0.120
Matawin	4.338**	0.000	3.665**	0.000	4.302**	0.000	11.78**	0.000	7.827**	0.000	3.212**	0.000
Vermillon	2.832**	0.009	2.613**	0.009	2.937*8	0.000	7.107**	0.000	3.649**	0.000	2.638**	0.000

** = significant Z value at the 5% threshold; * = significant Z value at the 10% threshold; Bold = significantly positive trend

**Table 5:** Results of the application of the various Mann–Kendall tests to the series of spring daily mean flows during the period 1930–2023

Rivers	MK		MMK-PW		TFPW		MMKY		MMKH		LTP	
	Z	*p* value	Z	*p* value	Z	*p* value	Z	*p* value	Z	*p* value	Z	*p* value
*Southeastern hydroclimatic region*												
Chateaugay	0.237	0.813	0.386	0.699	0.386	0.699	0.732	0.464	0.237	0.813	0.200	0.842
Eaton	− 1.863*	0.062	− *1.877*	0.061	− 2.10**	0.036	− 4.866**	0.000	− 1.863*	0.062	− 1.687*	0.093
Nicolet SW	− 1.464	0.143	0.162	− 1.272	− 1.272	0.203	− 3.683**	0.000	− 1.668*	0.095	− 1.559	0.119
Etchemin	− 1.414	0.157	− 0.905	0.366	− 1.258	0.208	− 3.632**	0.000	− 1.617	0.106	− 0.831	0.409
Beaurivage	− 0.579	0.563	− 0.358	0.720	− 0.358	0.720	− 1.355*	0.175	− 0.579	0.563	− 0.449	0.657
Du Sud	− 1.106	0.271	− 1.045	0.286	− 1.152	0.249	− 3.521**	0.000	− 1.101	0.271	− 0.727	0.470
*Eastern hydroclimatic region*												
Ouelle	0.656	0.512	0.616	0.538	0.775	0.438	1.631	0.103	0.656	0.512	0.421	0.674
Du Loup	− 0.418	0.676	− 0.266	0.790	− 0.351	0.726	− 0.909	0.363	− 0.418	0.676	− 0.298	0.770
Trois-Pistoles	0.850	0.395	0.670	0.503	0.854	0.393	1.672*	0.095	0.726	0.468	0.456	0.645
Rimouski	− 0.321	0.748	− 0.138	0.890	− 0.174	0.862	− 0.612	0.541	− 0.321	0.748	− 0.327	0.749
Matane	0.014	0.989	0.266	0.790	0.273	0.785	0.036	0.971	0.014	0.989	0.011	0.991
Blanche	0.492	0.622	0.626	0.531	0.907	0.364	0.388	0.698	0.340	0.734	0.127	0.899
*Southwestern hydroclimatic region*												
Petite Nation	3.722**	0.000	3.285**	0.001	3.710**	0.000	6.808**	0.000	4.620**	0.000	2.449**	0.014
Du Nord	1.031	0.302	0.897	0.369	0.768	0.443	2.605**	0.009	1.042	0.297	1.047	0.295
L’Assomption	1.422	0.155	1.655	0.098	1.464	0.143	3.779**	0.000	1.422	0.155	1.390	0.165
Matawin	1.650	0.098	1.768	0.077	1.596	0.110	3.440**	0.001	1.650	0.099	1.559	0.119
Vermillon	1.443	0.149	1.690	0.091	1.485	0.138	3.470**	0.001	1.316	0.188	1.323	0.186

** = significant Z value at the 5% threshold; * = significant Z value at the 10% threshold; Bold = significantly positive trend; Italic = significantly negative trend

**Table 6:** Results of the application of the various Mann–Kendall tests to the series of summer daily mean flows during the period 1930–2023

Rivers	MK		MMK-PW		TFPW		MMKY		MMKH		LTP	
	Z	*p* value	Z	*p* value	Z	*p* value	Z	*p* value	Z	*p* value	Z	*p* value
*Southeastern hydroclimatic region*												
Chateaugay	3.666**	0.000	3.321**	0.001	3.562**	0.000	7.718**	0.000	3.666**	0.000	3.357**	0.001
Eaton	1.568*	0.117	1.914*	0.056	1.840*	0.065	4.582**	0.000	1.568	0.117	1.434	0.152
Nicolet SW	1.854*	0.064	1.676*	0.094	1.953*	0.051	4.332**	0.000	1.854*	0.064	1.418	0.258
Etchemin	0.167	0.869	0.184	0.854	0.177	0.860	0.409	0.693	0.167	0.868	0.146	0.884
Beaurivage	− 0.301	0.763	− 0.047	0.963	− 0.032	0.974	− 0.799	0.424	− 0.295	0.768	− 0.245	0.811
Du Sud	− 0.753	0.452	− 0.705	0.481	− 0.719	0.472	− 1.760	0.078	− 0.635	0.525	− 0.547	0.588
*Eastern hydroclimatic region*												
Ouelle	− 2.349**	0.019	− 2.194**	0.028	− 2.110**	0.035	− 7.603**	0.000	− 2.349**	0.000	− 2.407**	0.016
Du Loup	− 2.364**	0.018	− 2.130**	0.033	− 2.070**	0.038	− 6.055**	0.000	− 2.364**	0.000	− 1.973*	0.049
Trois-Pistoles	− 2.056**	0.040	− 1.790*	0.074	− 1.804*	0.071	− 6.629**	0.000	− 2.056**	0.040	− 1.823*	0.069
Rimouski	− 3.611**	0.000	− 2.995**	0.003	− 3.484**	0.008	− 8.278**	0.000	− 3.814**	0.000	− 2.404**	0.016
Matane	− 2.895**	0.004	− 2.260**	0.024	− 2.671**	0.008	− 9.612**	0.000	− 2.640**	0.008	− 2.364**	0.018
Blanche	0.642	0.521	0.474	0.634	0.672	0.502	0.506	0.576	0.589	0.559	− 0.245	0.811
*Southwestern hydroclimatic region*												
Petite Nation	− 0.110	0.913	0.112	0.911	0.126	0.900	− 0.246	0.806	− 0.110	0.913	− 0.129	0.904
Du Nord	1.038	0.299	0.941	0.347	0.933	0.351	3.447	0.001	2.535	0.011	1.210	0.226
L’Assomption	− 0.878	0.380	− 0.776	0.438	− 0.734	0.463	− 3.726	0.000	− 1.911	0.056	− 1.018	0.313
Matawin	− 0.948	0.344	0.307	− 0.985	0.985	0.325	− 2.219	0.026	− 0.941	0.344	− 0.830	0.410
Vermillon	− 3.039**	0.002	− 2.881**	0.004	− 2.881**	0.004	− 8.114**	0.000	− 3.039**	0.002	− 2.954**	0.003

** = significant Z value at the 5% threshold; * = significant Z value at the 10% threshold; Bold = significantly positive trend; Italic = significantly negative trend

**Table 7:** Results of the application of the various Mann–Kendall tests to the series of fall daily mean flows during the period 1930–2022

Rivers	MK		MMK-PW		TFPW		MMKY		MMKH		LTP	
	Z	*p v*alue	Z	*p* value	Z	*p* value	Z	*p* value	Z	*p* value	Z	*p* value
*Southeastern hydroclimatic region*												
Chateaugay	4.724**	0.000	4.235**	0.000	4.523**	0.000	8.432**	0.000	4.724**	0.000	4.781**	0.000
Eaton	1.048	0.294	1.208	0.227	1.089	0.276	4.365**	0.000	1.160	0.246	1.288	0.198
Nicolet SW	2.165**	0.000	2.058**	0.040	1.979**	0.048	6.867**	0.000	2.003**	0.045	2.869**	0.004
Etchemin	2.339**	0.019	2.148**	0.032	2.068**	0.039	7.405**	0.000	2.339**	0.019	2.436**	0.015
Beaurivage	1.856*	0.063	2.376**	0.018	2.104**	0.035	6.438**	0.000	1.856*	0.063	2.727**	0.006
Du Sud	1.053	0.293	1.092	0.225	0.948	0.349	3.433**	0.001	0.970	0.332	1.344	0.179
*Eastern hydroclimatic region*												
Ouelle	0.097	0.922	− 0.073	0.942	− 0.095	0.924	0.298	0.766	0.099	0.921	0.145	0.885
Du Loup	− 1.070	0.284	1.180	0.238	− 1.019	0.309	− 4.727**	0.000	− 1.296	0.195	− 1.844	0.067
Trois-Pistoles	0.712	0.476	0.487	0.674	0.674	0.500	2.178**	0.029	0.712	0.476	0.778	0.437
Rimouski	1.171	0.241	0.256	1.034	1.034	0.301	3.412**	0.001	1.171	0.241	1.344	0.179
Matane	0.719	0.472	0.611	0.508	0.508	0.611	3.216**	0.027	0.913	0.361	0.697	0.496
Blanche	1.226	0.220	0.456	1.140	1.140	0.254	1.586	0.113	1.226	0.220	0.652	0.514
*Southwestern hydroclimatic region*												
Petite Nation	4.119**	0.000	4.245**	0.000	4.121**	0.000	11.76**	0.000	4.282**	0.000	5.624**	0.000
Du Nord	2.772**	0.006	3.153**	0.002	2.735**	0.006	9.606**	0.000	10.62**	0.000	5.864**	0.000
L’Assomption	1.998*	0.047	2.498**	0.013	2.195**	0.028	9.265**	0.000	7.026**	0.000	4.115**	0.000
Matawin	1.678*	0.093	1.735*	0.083	1.476	0.140	6.462**	0.000	2.273**	0.023	2.626**	0.000
Vermillon	2.378**	0.017	2.548**	0.011	1.950*	0.051	6.569**	0.000	9.571**	0.000	3.434**	0.000

** = significant Z value at the 5% threshold; * = significant Z value at the 10% threshold; Bold = significantly positive trend;

In the spring, the results of five of the six tests applied are concordant in the three hydroclimatic regions (Table 5): no significant change in the average flows. However, the results obtained by the MMKY method (which eliminates the effects of short-term persistence by correcting the variance of the series) revealed a significant generalized decrease in spring flows in the southeastern hydroclimatic region on the other hand, a significant generalized increase in these flows in the southwestern hydroclimatic region.

**Fig. 5:**
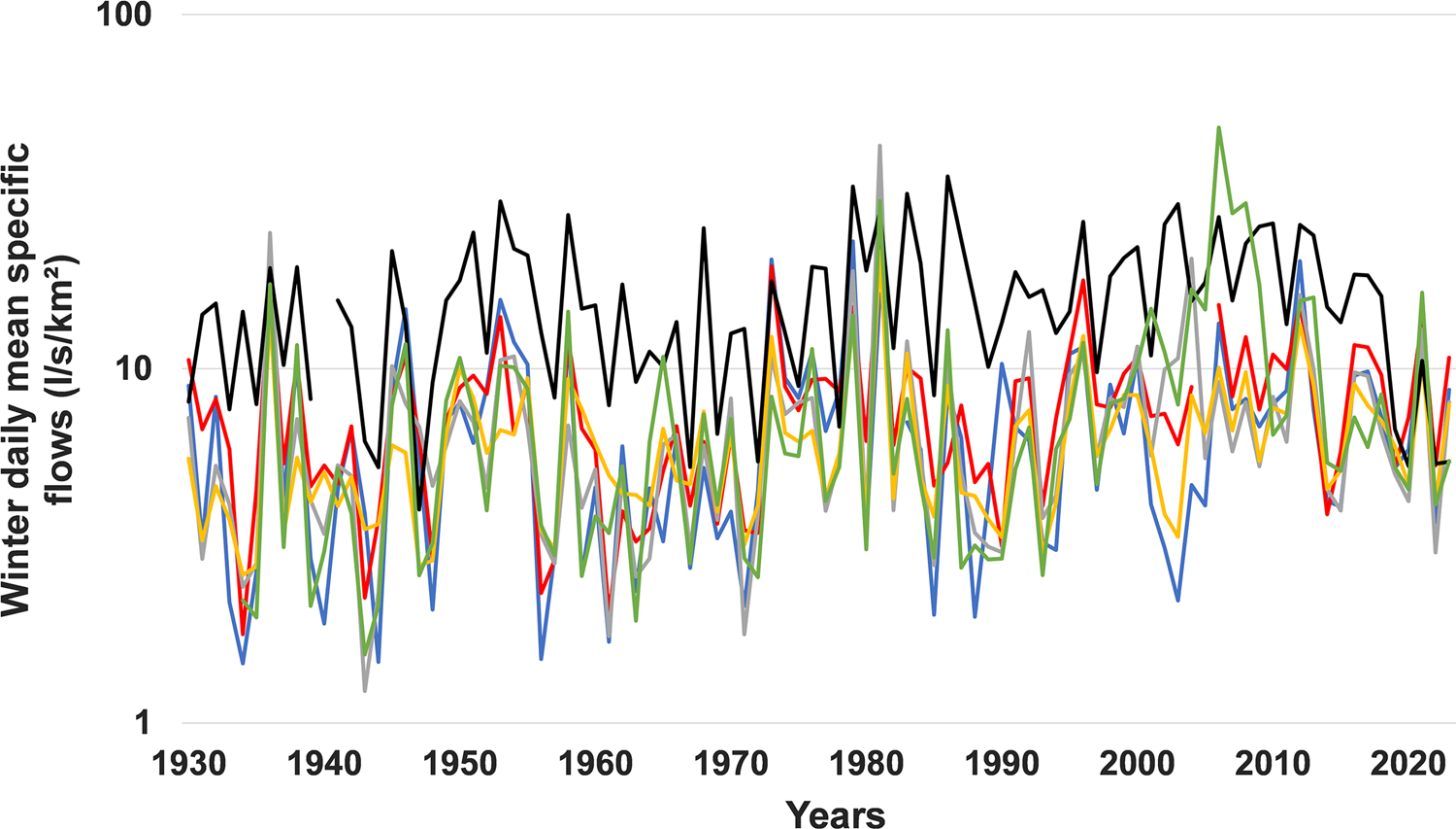
Interannual variability of winter daily minimum specific flows (l/s/km^2^) in the Eastern hydroclimatic region in south shore from 1930–2023. Ouelle River (SS7, Blue curve), Du Loup River (SS8, red curve), Trois-Pistoles River (SS9, gray curve), Rimouski River (SS10, yellow curve), Matane River (SS11, black curve), Blanche River (SS12, green curve)

**Fig. 6:**
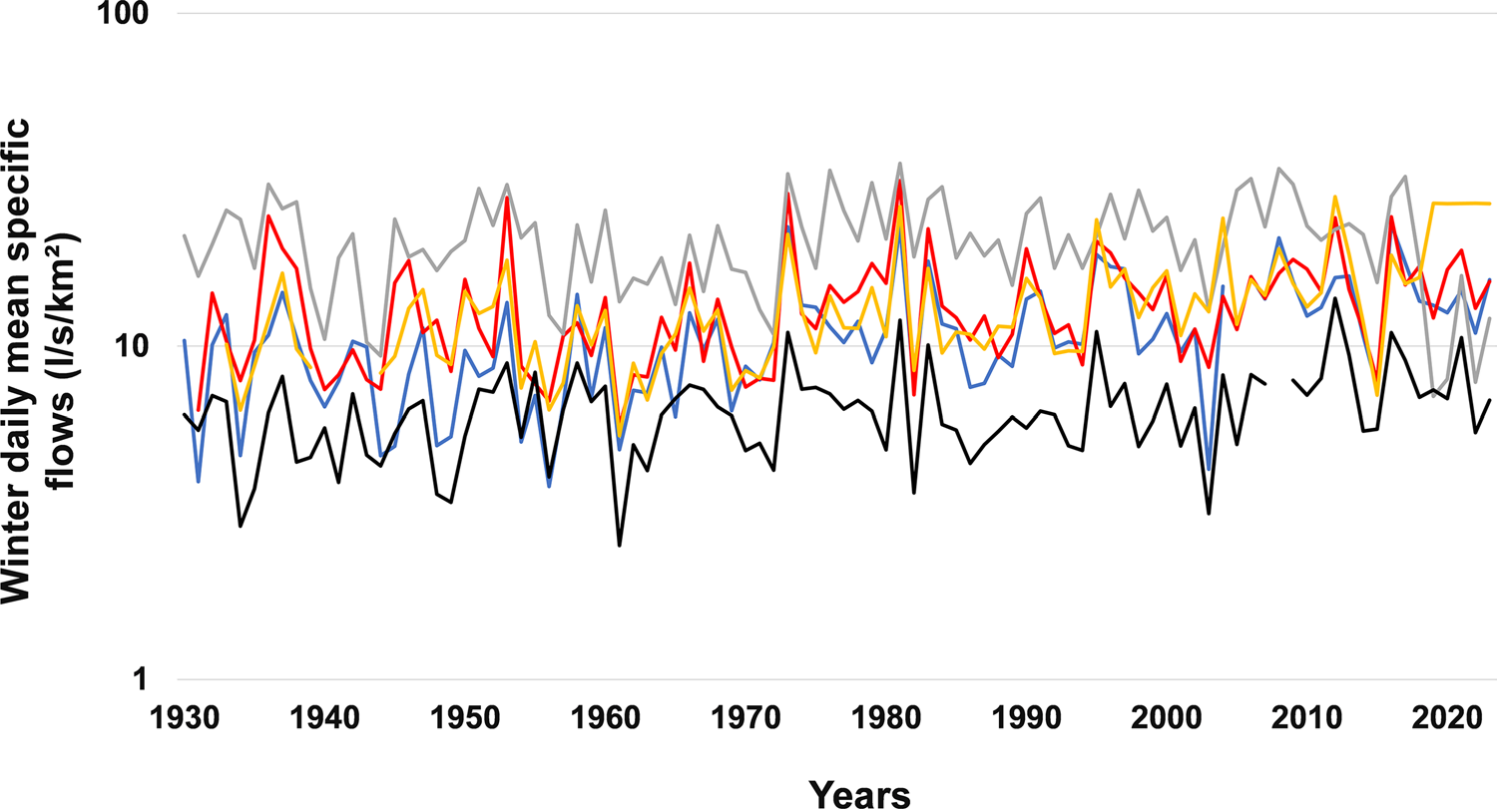
Interannual variability of winter daily minimum specific flows (l/s/km^2^) in the Southwestern hydroclimatic region in north shore from 1930 to 2023. De La Petite Nation River (NS1, Blue curve), Du Nord River (NS2, red curve), L’Assomption River (NS3, gray curve), Matawin River (NS4, yellow curve), Vermillon River (NS5, black curve)

In summer, the results of these six tests are almost consistent in the three hydroclimatic regions (Table 6). These tests revealed a significant almost generalized decrease in flows in the Eastern hydroclimatic region, with the exception of the Blanche River (Fig. 5). On the other hand, in the Southwestern hydroclimatic region, the significant decrease in flows was observed only for a single river (Vermilion River). As for the Southeastern hydroclimatic region, the tests detected a significant increase in the average flows for three rivers. Nevertheless, according to the LTP test, this increase is significant for only one river (Châteaugay). No significant decrease in flows was observed in this region (Fig. 6).

Finally, in fall, the results of these six tests agree with the hydroclimatic regions of the southeast and southwest. They show an almost general increase in flows in these two regions. In the hydroclimatic region of the east, five of these six tests revealed no significant change in the average fall flows over time.

## Discussion

### Analysis of the spatial variability of flows during the four seasons.

The correlation analysis demonstrated that the factors of the spatial variability of the seasonal average daily flows vary from one season to another. In winter, the flows are positively correlated with the rainfall and the maximum daily temperatures. These two climatic factors are closely auto-correlated. In fact, during this cold season, the occurrence of positive maximum temperatures is accompanied by an increase in precipitation in the form of rain; which promotes surface runoff supported by melting snow. Thus, the watersheds that experience a greater frequency of these occurrences have relatively high average daily winter flows. It is important to note that these winter flows are not correlated to any physiographic factor or to a land use or cover factor. This lack of influence of these factors can be explained by the fact that in winter, the ground being frozen, these factors cannot significantly affect the runoff process.

Spring flows are positively correlated with drainage density and snowfall. Drainage density influences the runoff process by transferring snowmelt water from slopes to river channels. The higher the drainage density in a catchment area, the greater this transfer of water, thus increasing river flows. As for the amount of snow accumulated on the ground, it influences the volume of water flowing into rivers because, in spring in Quebec, spring flows are mainly generated by snowmelt (Assani and Tardif 2005). Thus, the greater this quantity of snow, the higher the spring flows fed by the melting of this snow. On the other hand, the spring flows are on the other hand strongly negatively correlated with the maximum daily temperatures during the cold period, in particular those of April, the first month of the spring season. Relatively high temperatures promote rapid and early spring snowmelt. Thus, the amount of snow accumulated on the ground decreases very quickly throughout the spring season. This effect is amplified by rain (negative correlation between rain and spring flows). This rapid decrease in the amount of snow at the beginning of spring reduces the volume of water brought by snowmelt to the rivers, whose flows thus become relatively low for the rest of the season.

In a recent study, Assani (2022a) demonstrated that the magnitude of maximum daily spring flows from snowmelt are strongly negatively correlated with the area of wetlands and other water bodies due to the surface storage of runoff water fed by this snowmelt. In the scientific literature, this negative correlation has always been interpreted within the framework of the “sponge effect” concept (e.g., Acreman and Holden 2013; Lane et al. 2018) even in southern Quebec (e.g., Fossey and Rousseau 2016a, b; Fossey et al. 2015; Blanchette et al. 2019). However, Assani et al. (2023) demonstrated that it is not this concept but “storage surface” concept, a new concept which makes it possible to better explain the impacts of wetlands on the magnitude and duration of spring floods generated by melting snow in southern Quebec. Furthermore, with regard to the spatial variability of seasonal and annual daily minimum flows, Assani (2023b) and Assani et al. (2021, 2022) have clearly demonstrated that this spatial variability is mainly influenced by the agricultural area than by that of wetlands in southern Quebec during all seasons. These observations confirm that the sponge effect of wetlands plays only a minor role in this spatial variability of these minimum flows in southern Quebec (Assani 2022b).

In summer, average daily flows are positively correlated with average slopes of watersheds and forest area. It should be mentioned from the outset that in southern Quebec, the least agricultural watersheds have large areas of forest. These watersheds are mainly located on the north shore (southwestern hydroclimatic region) than on the south shore (southeastern and eastern hydroclimatic regions). However, the watersheds of the north shore, circumscribed almost entirely within the Canadian Shield, are characterized by higher average slopes than those of the most agricultural watersheds of the south shore which extend over the Lowlands of St-Lawrence, a geological formation characterized by a relatively flat topography. It follows that the average slopes of the basins are thus positively correlated with forest areas in southern Quebec. The impacts of forests on flows have already been the subject of numerous studies in the scientific literature. But there is still some controversy about these impacts (e.g., Alila et al. 2009; Andréassian 2004; De Walle 2003). In southern Quebec, to assess these impacts, a study was conducted in the Famine River watershed by Lavigne et al. (2004). The study analyzed these impacts under different scenarios of the decrease in forest area ranging from 1 to 72%. Under these different scenarios, deforestation caused a significant increase in the average daily spring and summer flows. The flows of two other seasons were not analyzed. Nevertheless, when this deforestation is coupled with agriculture, several studies have shown that in southern Quebec, the coupling of these two types of anthropogenic activity (deforestation and agriculture) causes a significant increase in flood flows, but, on the other hand, a significant drop in low water flows due to soil sealing caused by agricultural activities (Assani 2022b; Assani et al. 2016, 2021; Muma et al. 2011; Sylvain et al. 2015). However, on the hydrological level, the summer season corresponds to the period of low water, that is to say, to low water flows in Southern Quebec. It follows that the positive correlation observed between the forest area and the average daily summer flows, which mainly correspond to low water flows, is explained by the fact that the magnitude of these flows is lower in the most on the south shore with a smaller forest area than in the less agricultural watersheds on the north shore which, on the other hand, have a larger forest area. Thus, the influence of forest area actually reflects that of agricultural area. This reverses the impact of deforestation on the low water flows that characterize the summer season in Quebec.

Contrary to the average daily flows of three other seasons, the average daily flows in autumn are not correlated to any physiographic or climatic factor. However, Assani et al. (2021) observed a significant positive correlation between fall daily minimum flows and wetland area but negative with agricultural area. On the other hand, as for Assani (2024), he observed a significant negative correlation between the maximum daily autumn flows and the area of wetlands but positive with the agricultural area. Moreover, unlike the minimum flows, he observed a significant positive correlation between the maximum daily flows in the autumn and the summer and fall total rainfall. It follows that the lack of correlation between the average daily autumn flows and the land use and land cover factors does not seem, at first sight, to be explained by any rational factor. We can perhaps invoke the influence of evapotranspiration which can inhibit or attenuate the effects of physiographic factors and land use/cover on the spatial variability of daily flow in autumn, unlike daily maximum flows. This hypothesis is worth exploring.

### Analysis of the temporal variability of flows during the four seasons

The application of Mann–Kendall, four statistical tests that eliminate the effects of short-term persistence (SPT) and one test that eliminates the effects of long-term persistence (LTP) revealed that the variability of the average daily flows during the period 1930–2023 varies from one season to another on the one hand, and it is spatially heterogeneous, on the other hand, in southern Quebec.

In winter, the first five tests revealed a general increase in average daily flows in the three hydroclimatic regions of southern Quebec. However, this increase was only detected for one river in the southeastern hydroclimatic region by the last test (LTP). Be that as it may, we can admit that in winter, river flows have increased significantly over time in southern Quebec. This increase has already been observed by Beauchamp et al. (2015) and Assani et al. (2022) regarding the winter daily maximum and minimum flows in the three hydroclimatic regions. Three factors can be invoked to explain this increase in winter flows: (1) the early melting of snow due to the (2) increase in winter temperature observed in Quebec (Yagouti et al. 2008) and (3) the increase in precipitation in the form of rain. Early snowmelt in winter has already been observed in many regions of North America, notably (Burn 2008; Cunderlik and Ouarda 2009; Déry et al. 2009; Hodgkins and Dudley 2006) even in southern Quebec (Mazouz et al. 2012). As for the increase in precipitation in the form of rain in winter, the analysis of the temporal variability of the rainfall in winter in a few stations located in the three hydroclimatic regions during the period 1950–2020, carried out within the framework of this study, highlighted this increase (the results are not presented here) thus confirming the observations of Yagouti et al. (2008). In addition to these three factors, we must add another equally important one: the increase in rainfall in autumn. Remember that fall rainfall influence flows in winter by feeding groundwater and water levels in channels when precipitation falls exclusively in the form of snow, thus inhibiting any surface runoff. If the rains are relatively abundant in autumn, groundwater recharge will also be important and the water levels in the rivers will be relatively high, thus increasing the magnitude of the winter daily mean flows. However, as we will see later, the rainfall increased significantly in the fall in southern Quebec. This increase also causes that of the average daily flows in winter. Finally, this increase in flows in winter in the context of current climate warming has been predicted by all climate models in southern Quebec (e.g., Boyer et al. 2010).

On the other hand, in the spring, five of the six statistical tests applied revealed no significant change in the average daily flows in the three hydroclimatic regions. Only the MMKY test revealed an almost general decrease in flows in the southeastern hydroclimatic region on the south shore, the most agricultural, and a general increase in these flows in the southwestern hydroclimatic region on the north shore, the least agricultural. These results allow us to conclude that unlike winter flows, spring flows have not changed significantly over time in the three hydroclimatic regions of southern Quebec. It should be remembered that spring flows are generated mainly by the melting of snow accumulated in winter and spring. However, several studies have already shown that the snowfall in winter and spring has decreased significantly since the middle of the last century in southern Quebec due to the rise in temperature (e.g., Brown 2010; Guerfi et al. 2015; Yagouti et al. 2008), like many cold temperate regions (e.g., Danco et al. 2016; Janoski et al. 2018). Based on these observations, all climate models have predicted a significant decrease in spring flows in southern Quebec (Boyer et al. 2010). But this decrease in the snowfall does not translate into lower spring flows as predicted by these climate models. On the contrary, in the southwestern hydroclimatic region in particular, the flows even tend to increase over time despite a greater drop in the snow cover observed in this region (Brown 2010). This lack of decrease in spring flows despite the decrease in the snowfall is explained by an increase in the rainfall in the spring, which thus compensates for the decrease in the snowfall (Assani 2023; Assani et al. 2023). In fact, the analysis of the amount of rainfall in spring in several stations in the three hydroclimatic regions revealed an upward trend in this amount of rainfall.

In summer, two opposing trends clearly emerge. The first trend is characterized by an increase in daily mean flows. It is observed in the southeastern hydroclimatic region, the most agricultural. But this increase does not affect all the rivers in this region. As for the second trend, it is characterized by a significant decrease in flows over time. It is observed in the eastern hydroclimatic region on the south shore and the southwestern hydroclimatic region on the north shore. In the eastern hydroclimatic region, this drop in flow is almost generalized, while in the southwestern region, it seems to affect only a few rivers. Note that the LTP test only detected this drop for a single river in this latter hydroclimatic region. In the eastern hydroclimatic region, the general decrease in flows is explained by the decrease in the snowfall, the melting of which is the main source of groundwater in the spring. It is this hydroclimatic region that receives the greatest amount of snow than the other two climatic regions. Thus, the impact of the reduction in this snowfall on river flows is much more significant than in the other two hydroclimatic regions. Remember that summer flows are mainly fed by groundwater in southern Quebec.

Unlike the summer season, the fall season is characterized by a general increase in daily mean flows in the two hydroclimatic regions of the southeast and southwest. On the other hand, no change is observed in the eastern hydroclimatic region. This increase is explained by the increase in the rainfall observed in Quebec in particular (Assani 2024; Assani et al. 2008, 2012; Perrault 1999) and in the northeastern region of North America in general (e.g., Sadri et al. 2006; Small 2006). But this increase is not uniform across Quebec. It is greater in the first two regions than in the third hydroclimatic region (Assani 2024).

In their study devoted in particular to the temporal variability of monthly flows across Canada using the Mann–Kendall method—which eliminates short-term persistence (TFPW)—during the period 1947–1996, Zhang et al. (2001) observed an almost generalized decrease in flows from June to September but, on the other hand, an almost generalized increase in flows from March and April. When we compare these results with ours, we see that the reduction in flows was observed in summer (July to September) in two hydroclimatic regions, the least agricultural. The most agricultural hydroclimatic region (southeast) was characterized rather by an increase in summer flows. As for the month of March, it is part of the winter season which was characterized by an almost general increase in flows due to the early melting of snow. In contrast, the spring season, which includes the month of April in the context of our study, has no significant change in flows in the three hydroclimatic regions.

## Conclusions

The changes in precipitation and temperature regimes induced by the current global warming impact the different types of flows to varying degrees. These impacts have been widely analyzed on extreme flows in Canada and Quebec. It was therefore important to determine whether seasonal daily mean flows can be used to detect these climatic changes. Thus, an analysis of their spatiotemporal variability was undertaken to verify their sensitivity to global warming and their regional variability in southern Quebec.

The analysis of the spatial variability of the daily mean flows of four seasons revealed that, unlike the daily extreme maximum and minimum flows, these seasonal flows are very little influenced by physiographic factors and land use/cover. Their spatial variability is strongly influenced by climatic factors. But the extent of this influence varies seasonally. Thus, during the cold period, the average daily flows in winter are correlated with rainfall and daily maximum temperatures (positive correlation), two factors that influence snowmelt. In spring, these flows are strongly influenced by the snowfall (positive correlation) and at winter and spring daily maximum temperatures (negative correlation). These two factors influence the volume of surface runoff and the rate of spring snowmelt. During the warm period, in the summer season, the spatial variability of the average daily flows is slightly influenced by the rainfall (positive correlation) which generates surface runoff. On the other hand, in autumn, the flows are no longer significantly correlated with any climatic factor.

As for the temporal variability of seasonal daily mean flows, it shows clear seasonal and regional differences. In winter, the application of six different statistical tests that eliminate short-term (STP) and long-term (LTP) persistence effects to flow data measured from 1930 to 2023 revealed an almost generalized increase in flows in the winter in the three hydroclimatic regions of southern Quebec due to the increase in fall and winter rainfall as well as the winter temperature that causes the early snowmelt. On the other hand, in spring, no significant change in flows over time was observed despite the decrease in the snowfall observed since the middle of the last century. In summer, the striking fact is the decrease in flows over time. This decrease is more generalized in the eastern hydroclimatic region on the south shore than in the other two hydroclimatic regions. It would result from the decrease in the snowfall. Finally, in autumn, the increase in flows is generalized in the hydroclimatic regions of the southwest and southeast due to the significant increase in the rainfall. This increase is less significant in the eastern hydroclimatic region where flows have changed little over time.

This study demonstrates that seasonal daily mean flows are as sensitive as extreme flows to changes in temperature and precipitation regimes. They can therefore be used to detect and monitor the impacts of global warming on the evolution of flows in southern Quebec. But the degree of this sensitivity depends on the seasons. The two most sensitive seasons are winter and autumn due to the rise in temperature and rainfall. Thus, the effects of the decrease in the snowfall on the seasonal average daily flows seem limited to southern Quebec due to the increase in rainfall. The study also reveals that the hydrological impacts induced by this global warming vary according to hydroclimatic regions, an aspect that is not generally taken into account by climatic and hydrological models to predict changes in flows in the coming decades. Thus, in the problem of water management in southern Quebec in the context of current global warming, it is imperative to take into account these regional hydroclimatic differences.

## Data Availability

The author may provide the data used in the manuscript upon request.
